# Health Chatbots in Africa: Scoping Review

**DOI:** 10.2196/35573

**Published:** 2023-06-14

**Authors:** Millie Phiri, Allen Munoriyarwa

**Affiliations:** 1 School of Communication University of Johannesburg Westcliff South Africa

**Keywords:** chatbots, health, Africa, technology, artificial intelligence, chatbot, health promotion, health database, World Health Organization, WHO, rural area, epidemiology, vulnerable population, health sector, Cochrane database

## Abstract

**Background:**

This scoping review explores and summarizes the existing literature on the use of chatbots to support and promote health in Africa.

**Objective:**

The primary aim was to learn where, and under what circumstances, chatbots have been used effectively for health in Africa; how chatbots have been developed to the best effect; and how they have been evaluated by looking at literature published between 2017 and 2022. A secondary aim was to identify potential lessons and best practices for others chatbots. The review also aimed to highlight directions for future research on the use of chatbots for health in Africa.

**Methods:**

Using the 2005 Arksey and O’Malley framework, we used a Boolean search to broadly search literature published between January 2017 and July 2022. Literature between June 2021 and July 2022 was identified using Google Scholar, EBSCO information services—which includes the African HealthLine, PubMed, MEDLINE, PsycInfo, Cochrane, Embase, Scopus, and Web of Science databases—and other internet sources (including gray literature). The inclusion criteria were literature about health chatbots in Africa published in journals, conference papers, opinion, or white papers.

**Results:**

In all, 212 records were screened, and 12 articles met the inclusion criteria. Results were analyzed according to the themes they covered. The themes identified included the purpose of the chatbot as either providing an *educational* or information-sharing service or providing a *counselling* service. *Accessibility* as a result of either *technical* restrictions or *language* restrictions was also noted. Other themes that were identified included the need for the consideration of *trust*, *privacy and ethics*, and *evaluation.*

**Conclusions:**

The findings demonstrate that current data are insufficient to show whether chatbots are effectively supporting health in the region. However, the review does reveal insights into popular chatbots and the need to make them accessible through language considerations, platform choice, and user trust, as well as the importance of robust evaluation frameworks to assess their impact. The review also provides recommendations on the direction of future research.

## Introduction

Reviewing how health chatbots are being used in Africa is important for their future expansion in Africa. Public perception and—crucially—trust of chatbots are extremely influential. Various studies have indicated that understanding the impact of chatbots is problematic even in regions outside Africa and in sectors other than health [1]. However, high-quality data on quantitative and qualitative aspects of health outcomes and user experience must be considered to understand the value of these tools for Africa—a context where they are increasingly being deployed as internet access and connectivity improves yet are currently underresearched. This review contributes to knowledge in major ways: it identifies the scope of the deployment of chatbots and, in the process, provides evidence of the current state of knowledge in the field of health chatbots research. Knowledge of what research has been done and what the information gap needs are part of understanding the health chatbot terrain in Africa. It is for this reason that understanding future trends in chatbot use in Africa is pertinent and discussed in this review. Knowing where chatbots are currently deployed is pivotal to support the analysis of which areas are popular and which areas may need attention in the future.

Chatbots are computer programs designed to simulate human text or verbal conversations through the use of machine learning to make responses seem more humanlike [2]. They come in the form of voice, text, or images, which make them a powerful tool for disseminating health-promotion information. The basic concept of chatbot technology is not entirely new in Africa, although its adoption seems to have progressed slowly since the 1980s and has picked up in the last 6 years from 2017 onward. A literature review shows that the first medical artificial intelligence (AI) technologies were piloted in Africa in the 1980s, in countries including Kenya, Egypt, Gambia, and South Africa [3]. However, their use has become more pronounced since the pandemic and epidemic responses. Since 2020, the COVID-19 pandemic has globally magnified the importance of chatbots as a critical source of reliable information in public health management [4,5]. Research from 2020 to date shows the intensity in which researchers have explored the area of AI, including chatbots articulating the multipurpose role they play as communication tools, such as providing more understanding of diseases, being sources of reliable information, reducing exposure to infections, counselling, assisting health care workers with rapid and accurate solutions, and communicating with doctors, among other functions.

According to the World Health Organization (WHO), during the pandemic—where even some of the most resilient health systems were overwhelmed—people have turned toward digital channels for accessing information and advice for their health [6]. In Africa, WhatsApp chatbots in South Africa, Rwanda, and Senegal provided reliable information and support for rapid COVID-19 testing [7]. In Ghana, a free COVID-19 Telegram app chatbot was developed to tackle misinformation and improve access to accurate data [8]. However, the digital divide in Africa is real, and although Africa’s mobile phone market is growing, access is impeded by challenges such as limited internet access and connectivity, high data costs, low literacy levels, and inadequate power supplies [3].

This review sought to answer the following questions:

1. What does the literature on chatbots in Africa tell us about the design, uses, and user experience of this technology in this region’s health care sector?
2. To what extend can this literature inform us about policy issues, accessibility, and ethical and regulatory matters regarding chatbots in health care systems and services in Africa?
3. Where are the innovative measurement and evaluation frameworks emerging from and in what form regarding chatbots use in Africa’s health care?
4. What insights does the literature provide, if any, about emerging issues or the future of the chatbot landscape in Africa’s health care sector?

## Methods

### Overview

The scoping review used an approach based on the 2005 Arksey and O’Malley [9] framework. This commonly cited approach emphasizes that a scoping review should be able to (1) identify available evidence in a given field, (2) clarify concepts or definitions in the literature, (3) examine research conducted on a particular topic, (4) identify key characteristics or factors related to a topic, (5) be conducted as a precursor to a systematic review, and (6) identify and analyze knowledge gaps [10]. This review was particularly more interested in points 1, 3, 4, and 6, which we viewed as being the most appropriate to determine the scope and literature coverage of health chatbots in Africa.

### Search Criteria

Using a Boolean search that combined the terms “chatbots” OR “virtual assistant” OR “conversational agents” OR “AI enabled platform” AND “digital human” with the terms “health” AND “Africa,” a broad literature search was conducted between June and July 2021 and again in July 2022, looking at literature published between 2017 and July 2022. This period was important for the increased use of AI and chatbots on the continent due to increased technology use, particularly smartphones [5]. This approach was appropriate for a scoping review such as this one as Munn et al 2018 remind us that a scoping review is a tool that helps to focus on the literature of a topic and a new approach to synthesize evidence [10]. Literature was identified using Google Scholar, EBSCO information services—which includes the African HealthLine, PubMed, MEDLINE, PsycInfo, Cochrane, Embase, Scopus, and Web of Science databases—and other internet sources (including gray literature). This search included peer-reviewed academic journals, reviews, conference papers, and white papers.

### Inclusion and Exclusion Criteria

No language restrictions were applied to the literature search. The inclusion criteria were that the literature had to substantially focus on Africa or countries in Africa and be predominantly about health chatbots. The document had to be a journal paper, conference paper, opinion article, or white paper. Literature that focused on the broad subject of AI technologies was only considered relevant and included if health chatbots were the main feature of the study. Once the capturing process was done, the records were screened to ensure its relevance. Literature that mentioned health chatbots and Africa in passing or were broadly about AI was excluded. The exclusion criteria were any literature that was conducted outside of Africa or referred to non–health-related chatbots and nonresearch or nonconference papers.

### Screening, Data Extraction, and Analysis

This process of screening, data extraction, and analysis consisted of 3 stages done by 1 researcher (MP). The researcher conducted the first stage, which was to screen the identified records to ascertain if they were an academic research paper, review, conference paper, white paper, or none of these and if they mentioned Africa or health chatbots. The second stage was to further screen the results by closely examining if they were indeed about health chatbots in Africa. The third stage was to categorize the results as being relevant, partially relevant, or not relevant. An article was considered partially relevant if it related to health chatbots but used Africa as a reference point only, or it was about broader AI use and chatbots were not the main feature of the study. The final stage was done by the researcher and a reviewer (AM) and was used to fit the literature into research question themes and to look out and record new emerging themes using the thematic analysis approach.

## Results

### Overview

**Figure 1:**
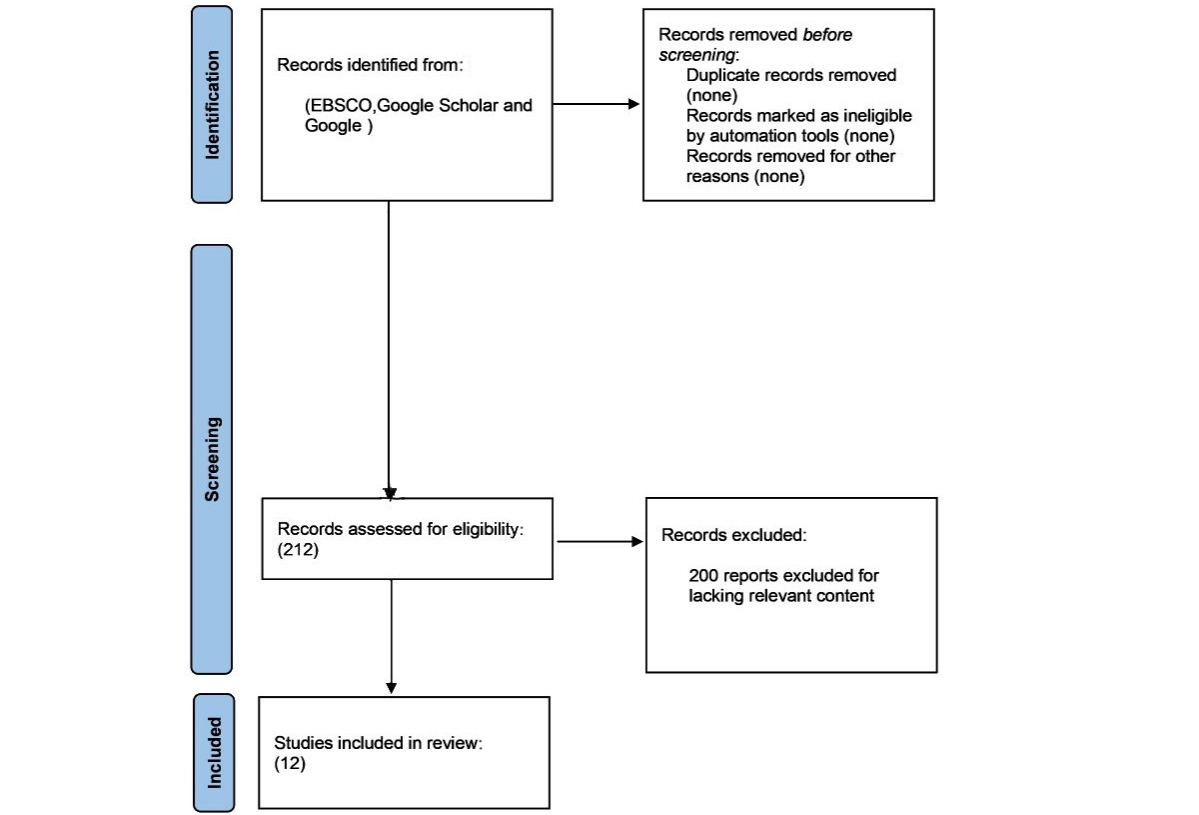
PRISMA (Preferred Reporting Items for Systematic Reviews and Meta-Analyses) diagram.

The initial search identified 212 articles, with no duplicates. After screening, 200 articles were excluded, and 12 studies were included (see Multimedia Appendix 1 [11-22] for the full list). Although 66 of the 200 excluded reports mentioned Africa or chatbots in passing, they did not address the chatbots in Africa to a sufficient extent (see Figure 1). Of the 12 identified studies, 6 (50%) focused on South Africa, 1 (8%) on Kenya alone, 1 (8%) on Kenya and Nigeria, 1 (8%) on Tunisia and Nigeria, 1 (8) on Nigeria alone, and 2 (17%) on the broader African continent. Five (42%) were peer-reviewed journal articles, 3 (25%) were preprints (or papers posted to open access web-based repositories), and 4 (33%) were conference papers. Many of the articles used mixed methods (both quantitative and qualitative) approaches to assess or evaluate chatbots. Some (n=4, 33%) of the articles described specific chatbots and their development, whereas 3 (25%) were reviews or narrative reviews. Four (33%) papers were focused on interventions addressing HIV, whereas another 4 (33%) looked at interventions that sprung from needs generated from the COVID-19 pandemic, including an intervention to address vaccine hesitancy and one to help people with type 2 diabetes during the pandemic. For example, a study in Nigeria showed that the majority of the apps or chatbots were focused on sex education (39.6%), sexually transmitted infection (8.6%), and HIV or AIDS testing (13.7%) [11]. The other studies were concerned with maternal care (n=3, 25%), general medical support (n=1, 8%), and psychological or mental health assessment or support (n=2, 17%).

Our findings show that research specifically focusing on chatbots in Africa is limited. The research that does exist mostly centers around infrastructure, uses, and user experience—particularly on the design of chatbots that use Indigenous languages or infrastructure that is user-friendly in Africa [12-14]. For issues pertaining to reach and regulation, research is anchored in issues such as the role of mobile phones in improving access to health services and on the technology’s ethical implications, such as confidentiality and security [15,23]. Chatbots are viewed as interventions that are potentially highly useful for improving access to health services and information, including support for health concerns and health-promoting behaviors such as fitness.

### Themes

#### Educational Chatbots

Several of the chatbots identified help provide patient information as well as offer behavior change in sex education, perinatal depression, and HIV. They provide more educational information on topics such as contraception, testing, and medication knowledge. For example, Kenya’s Zuri chatbot was programmed to walk a patient through a structured curriculum such as the Thinking Healthy program [16], whereas a Facebook Messenger chatbot was used to increase vaccination acceptance [17]. As a safety measure, conversations with patients in need of additional support can be handed over to live counselors as needed [16]. WhatsApp chatbots are mentioned as being important for interactive education, risk assessment, referrals, and contact tracing [23]

#### Counselling and Testing

Chatbots have been used in counselling and testing, particularly for HIV. In one study, participants indicated that talking to the chatbot agent felt “natural” and made them comfortable [18]. Some key concerns included the language the chatbot used, the overly rapid speed at which the agent responded, and the chatbot agent either misunderstanding or not understanding the questions. Users were happy with the confidentiality of the process, which was considered more private, anonymous, and avoided the need for them to visit a public health facility in person. A study in Nigeria found that 13% of chatbots reviewed were supporting HIV testing [11], whereas another found that it was useful to have chatbots on platforms that younger age groups use, indicating their potential to reach younger demographics [19].

#### Indigenous Languages

Relatively few chatbots specifically focused on the use of Indigenous languages and African dialects. Those that did were used in the context of malaria, tuberculosis, maternal health, and COVID-19. For example, the isiXhosa chatbots [13] in South Africa were used to answer maternal health questions and in crisis communication such as COVID-19. In one case, the proposed uses of the Likita chatbot [12] included diagnosing, scheduling appointments, offering treatments, and answering questions, but the idea has yet to be tested. A chatbot tested in Tunisia answered questions using English, French, Arabic, Tunisian, Igbo, Yoruba, and Hausa without any predefined scenarios, with results indicating user satisfaction [14]. In this case, the chatbot’s architecture could be tailored to any tokenized African dialect. However, this has not been used to its full potential in expanding language access. In general, there is a need to design chatbots that either already have or can be adapted to have multiple underrepresented African languages and dialects. Some of their inherent value as communication devices should be in the breadth of language options included in their content.

#### Linguistic Accessibility

The health chatbot landscape is sometimes hindered by language considerations. For a region considered to be home to roughly a third of the world’s languages, there has been limited research on chatbot solutions using local African languages. Language barriers have the potential to be a major issue for the region, especially since they are likely to disproportionately affect access for lower-income or isolated communities and may also reflect a lack of contextualization or understanding of local needs.

The review’s findings reveal that the future of chatbots in Africa depends to a large extent on deploying technologies that have an African perspective. This was shown in research on chatbots such as the Likita chatbot [12], the isiXhosa chatbots in South Africa [13], Mbaza in Rwanda [24], Zuri in Kenya [16], and an AI-powered chatbot for crisis communication (such as COVID-19) developed in Tunisia [14]. The architecture used by this last bot can be tailored to any African dialect, leaving room to design chatbots for other underrepresented African languages and dialects. These chatbots help improve access to information in their home language for the African people who need it most. It makes them feel comfortable, understood, and appreciated [13].

#### Technical Accessibility

Language is not the only consideration for accessibility. Internet connectivity and platform preferences also have a substantial impact. For instance, many advanced technologies require smartphones and internet access, which are associated with high data costs and inadequate power supplies [4]. Despite the growth of the smartphone market in Africa, many people are still excluded from accessing apps that are only available on smartphones. Even where access is hypothetically available, platform preferences and familiarity will also influence uptake.

That said, where access is possible, chatbots may well be a useful channel. A study from South Africa looking at how young people access HIV treatment services noted that access improved when young people had access to HIV prevention methods from platforms where they can engage in a nonintrusive way [10]. Young people are considered the largest market for cell phones in Africa, and they are also a group that would benefit from better access to health information, particularly information that might be culturally difficult to discuss with older adults such as sexually transmitted diseases, sex education, or HIV and AIDS. This indicates that health chatbots could potentially increase access to health services if the right technologies and platforms were used. However, it also highlights the need for more research on the platforms preferred by different demographics.

#### Trust

The findings also reveal that trust issues are extremely influential in the uptake and use of health chatbots. People tend to get information from sources they trust the most. This is in conformance with lessons learned throughout dealing with pandemics and epidemics such as HIV, Ebola, and COVID-19 that show that compliance with public health recommendations is a function of trust. In this review, there is evidence that one gains trust by helping audiences understand: by engaging with them, giving or acting on feedback, and making communities part of the solution. In Kenya, the Zuri health chatbot works by engaging a patient via a variety of trusted channels, including SMS text messaging [16]. Dispensing accurate, reliable information in real time is thus a way of enhancing the accessibility of health chatbots. The review shows that promoting trust is thus key to increasing use and accessibility to those who require the information the most.

Trust is also built through clear regulatory mechanisms that ensure stakeholders, especially patients, can receive trusted and real-time information [25]. This was the case with the Hello Chatbot [26], an app that allows users to communicate with health care providers, receive daily health advice, and access free health care information on demand. However, the review also found that studies about regulations are very scarce, which is potentially linked to the absence of national digital health strategies in many countries [4].

Finally, chatbot quality also affects trust. A study that aimed to review and identify high-quality HIV and AIDS mobile apps and chatbots to aid the development of effective and efficient coronavirus mobile apps and chatbots for use in resource-limited countries revealed that only about 12% of those available for use in Nigeria were of high quality according to the study definition [11].

#### Privacy and Ethics

Literature on the ethical implications for using health chatbots in Africa is not readily available. However, studies looking at chatbot use in HIV testing and counselling and for information on sexual health showed that privacy and confidentiality are important features that may promote the acceptability and willingness to use internet-enabled HIV prevention methods such as chatbots, especially for younger age groups [11]. The marked absence of these privacy features may in turn be affecting current chatbot use: a study on female sex workers in South Africa cited privacy concerns about using chatbots with respect to their potential disclosure of venues or to other people [20]. More research on the presence and perception of ethical issues would help to fill an important gap in the discourse about chatbots in the region.

#### Evaluation

Evaluation is a critical component of any health intervention, and chatbots are no exception. The findings reveal a strong leaning on evaluation research to assess the impact of health chatbots in the region. Evaluation is understood to be important in measuring the functionality, efficiency, and relevancy of existing chatbots and understanding their potential for further development or expansion. For example, a study on the Kenyan chatbot Zuri was used as an evaluation tool, and the results were ultimately used to refine the intervention content and add Swahili-language support [16]. Another study examined web-based conversations with chatbots to test the intelligence of a machine in textual conversations through a comparison with a human to test its “humanness” [27].

## Discussion

### Principal Findings

These results demonstrate that published literature on chatbots in Africa’s health space is scarce. The paucity of studies from Africa highlights a disparity in global research about chatbots and the health sector. This is despite the fact that Africa is an emerging market for chatbot use due to its relatively young population and a growing mobile phone sector [28]. The evidence does not show that this potential is being fully utilized in the field of health. Although chatbots are increasing in popularity, they are not a predominant research theme.

This review demonstrates where health chatbots in Africa have made positive impacts and where the gaps lie. Although this review was able to identify gaps, there remains a need for in-depth localized studies of the chatbots currently in use for a broader understanding of the continent’s health chatbots landscape in individual countries. The strength of this review has been its contribution to providing insights on trends within the region. An analysis of the data examined shows how accessibility, including language; ethical considerations; and trust are crucial parts of understanding health chatbots.

### Comparison to Prior Work

There is very limited prior work in this area. However, our analysis is in agreement with other reviews of the global literature that demonstrate the paucity of high-quality research on the effectiveness of chatbots as public health interventions [29]. The quantity of literature is increasing, particularly since the COVID-19 pandemic created a need for outreach that was not previously experienced. However, there is now a need for careful reflection on what populations need from chatbots and how they can be delivered in a safe and ethical manner [30].

### Gaps and Directions for Future Research

As noted, there are very few studies on health chatbots and user experience in Africa, particularly for rural areas where most African people live. This is despite research indicating the potential of new digital technologies to increase access to health services for rural populations or reduce the need for hospital visits, such as by assisting with triage before medical consultation. Chatbots have been identified as being potentially useful in diagnosing diseases such as malaria, where rural areas in particular face a shortage of skilled health workers and medical equipment [4].

Understanding whether ethics are adequately assessed and regulated in Africa is fundamentally important. Not much is known about the ethical codes and regulations that work best for the region, in part because there are complex attitudes surrounding the diagnosis for many diseases, which may affect the risk profile of sharing data or personal information on chatbots. It has been observed that “many people experience shame and marginalization due to a diagnosis and go on to describe the consequences of stigma as more burdensome than those of the condition itself” [31]. Developing stronger and clearer ethical codes may increase accessibility of services, but whether this will definitively change use trends in Africa is still largely unknown.

Future research directions that should be a priority for the field include:

- The need to further understand the use of voice chatbots and the integration of African dialects and languages into chatbot systems;
- The development of chatbots that can be integrated into smartphones;
- The identification of user needs and ensuring that chatbots specifically respond to these needs in both content and design;
- New evaluation frameworks for health chatbots and other emerging technologies;
- The linkage between web-based and offline services and how to avoid creating or exacerbating issues of equity in health service access;
- Literature on chatbots use in medical sectors other than HIV, sexual and reproductive health, maternal health, malaria, tuberculosis, and COVID-19;
- Literature on gaming health chatbots—the review only showed one such health chatbot that uses gamification in South Africa to reduce anxiety and stress and increase motivation, loyalty, and efficiency, especially in corporate environments [21]; and
- In particular, research on chronic disease health chatbots such as the Cape Town diabetes chatbot [22].

### Limitations and Strengths

One limitation of our search strategy was that if an article did not use the term “Africa” or any of the alternative names for chatbots that were used in this search, it may mean that those results were omitted. Africa is made up of 54 countries, and if “Africa” was not among the key words included in the search engine optimization, nationally relevant examples may not have been captured. However, a search was later conducted in PubMed that included the names of the 54 countries, and no additional records were identified.

### Conclusions

Across the continent of Africa, chatbots are already being used as a tool for health information and are growing in popularity. However, the findings show that many gaps remain in our understanding of their use in different countries; in key locations, especially rural areas; and among different user groups, especially vulnerable ones. Their use also appears to have been limited to specific disease areas such as malaria, HIV, maternal health, and sexual reproductive health; very little is known about their use or potential in other health disciplines. In a region with a fast-growing population and growing access to technology, more research should be conducted to better understand the true potential of chatbots to support health and well-being—both in terms of who they can reach and who they perhaps cannot. This research provides a scoping analysis of the deployment of chatbots. It lays the groundwork for future research in this field. For instance, future research can focus on the types of health information that users receive from chatbots.

## Appendix

Multimedia Appendix 1 Included studies of health chatbots in Africa.

## Abbreviations

AI: artificial intelligence
WHO: World Health Organization
