# Supplementary material for: Health Chatbots in Africa: Scoping Review
Source: J Med Internet Res. 2023 Jun 14;25:e35573. doi: 10.2196/35573 (PMC10337242; doi:10.2196/35573)
Supplement: Multimedia Appendix 1 [file jmir_v25i1e35573_app1.docx]

Multimedia index

|  | Relevant data | | | | | |
| --- | --- | --- | --- | --- | --- | --- |
| **Author name** | **Title** | **Date published** | **Country/Region** | **Subject area** | **Research Design** | **References** |
| 1.Miracle Adesina | 5.Learning from digital health in curtailing coronavirus epidemic: review of HIV/AIDS mobile apps or chatbots in Nigeria | 27 January -4 February | Nigeria | Reviews and identifies and rates the quality of HIV/AIDS mobile applications and chatbots available to Nigeria | Review | Conference Paper [11](https://www.researchgate.net/publication/349055334_Learning_from_digital_health_in_curtailing_coronavirus_epidemic_Review_of_HIVAIDS_mobile_apps_or_chatbots_in_Nigeria) |
| 2.Yaseerah Akoodie | 9.Gamification in psychological assessment in South Africa: A narrative review | January 2020 | South Africa | The use of gamification (including AI based gamification) in psychological assessment | Narrative review | Peer-reviewed Journal Article [21](https://doi.org/10.4102/ajopa.v2i0.24) |
| 3.Eric P Green | 6.Expanding access to perinatal depression treatment in Kenya through automated psychological support: development and usability study | October 2020 | Kenya | Prepilot study on the Healthy Moms perinatal depression intervention | Single-case experimental | Peer-reviewed Journal Article [16](https://www.ncbi.nlm.nih.gov/pmc/articles/PMC7573703/) |
| 4.Aymen Ben Elhaj Mabrouk | 3.A multilingual African embedding for FAQ chatbots | March 2021 | Tunisia and Nigeria | The development of an Artificial Intelligence Powered chatbot for crisis communication | Descriptive with some qualitative evaluation | Preprint: [14](https://arxiv.org/pdf/2103.09185.pdf) |
| 5.Robert Mash | 11.Evaluating the Implementation of the GREAT4Diabetes WhatsApp chatbot to educate people with type 2 diabetes during the COVID-19 pandemic: convergent mixed methods study | April/June 2022 | South Africa | Evaluates implementation of WhatsApp chatbot in Cape Town to manage diabetes during COVID-19 pandemic | Descriptive and qualitative | Peer-reviewed Journal Article [22](https://diabetes.jmir.org/2022/2/e37882) |
| 6.Amit Mishra | 2.Chatbot in isiXhosa for remote pre/post-natal care | July 2019 | South Africa | The development of chatbots in indigenous languages is new and still developing. | Descriptive | Preprint: [13](https://doi.org/10.31224/osf.io/jm54z) |
| 7.Kevin Mugoye | 4.Smart-bot technology: conversational agents’ role in maternal healthcare support | July 2019 | Africa | Discusses the need to develop chatbots to provide support to to-be mothers during their journey in pregnancy. | Narrative review | Conference Paper: [15](https://ieeexplore.ieee.org/document/8764817) |
| 8.Oladapo Oyebode | 1.Likita: A medical Chatbot to improve healthcare delivery in Africa | April 2018 | Africa | Describes an intelligent medical chatbot | Descriptive | Conference Paper: [12](http://dx.doi.org/10.13140/RG.2.2.29159.09128) |
| 9.Leah Rosenzweig | 12. Testing interventions to address vaccine hesitancy on Facebook in  East and West Africa | January 2022 | Kenya, Nigeria | Evaluates the extent to which chatbot conversation is effective at increasing vaccination acceptance and self-reported vaccine intentions | Quantitative and qualitative | Preprint: [17](https://osf.io/vjk6z) |
| 10.G Tshabalala | 8.Developing a chatbot for HIV risk assessment among young people living in Soweto, South Africa | January 27 to 4 February 2020 | South Africa | Use of chatbots to promote confidentiality among young people living positively with HIV | Qualitative | Peer-reviewed Journal Article [19](https://onlinelibrary.wiley.com/doi/10.1002/jia2.25659) |
| 11.Alastair van Heerden | 10. The potential of conversational agents to provide a rapid HIV counselling and testing services | October 2017 | South Africa | User experience of a conversational agent providing HIV  counseling and testing session. | Quantitative (n=10) | Conference Paper: [18](https://sci-hub.se/10.1109/FADS.2017.8253198) |
| 12,William X You | 7.Facilitators and barriers to incorporating digital technologies into HIV care among cisgender female sex workers living with HIV in South Africa | April 2020 | South Africa | Exploring the use of digital technologies to deliver HIV-related interventions to female sex workers | Qualitative | Peer-reviewed Journal Article [20](https://pubmed.ncbi.nlm.nih.gov/32270007/) |
